# Optical Sensing Properties of Dithiocarbamate-Functionalized Microspheres, Using a Polyvinylpyridine-Polyvinylbenzyl Chloride Copolymer

**DOI:** 10.3390/s101008953

**Published:** 2010-10-08

**Authors:** Ziad M. Shakhsher, Imad M.A. Odeh, Inas M.S. Rajabi, Mahmoud K. Khatib

**Affiliations:** Chemistry and Chemical Technology Department, College of Science and Technology, Al-Quds University, Jerusalem, Israel; E-Mails: zshakhsher@science.alquds.edu (Z.S.); RajabiI@science.alquds.edu (I.R.); mkakb2001@yahoo.com (M.K.)

**Keywords:** optical sensor, dithiocarbamate-copolymer, polymer swelling

## Abstract

In this study, a new modified optical chemical sensor based on swellable polymer microspheres is developed using a 5% copolymer of polyvinylpyridine-polyvinyl-benzyl chloride microspheres functionalized as the corresponding dithiocarbamate. This sensor demonstrated significant enhancements in sensitivity, dynamic range and response time. These improvements are related to the presence of pyridine in the polymer backbone, which is believed to increase the space between the groups, thus decreasing steric hindrance, and hence increasing substitution of the dithiocarbamate group. The hydrophilicity of pyridine also allows free movement of the solvent and analyte to and from the inside of the microspheres. These dithiocarbamate-derivatized polymer microspheres were embedded in a hydrogel matrix of polyvinylalcohol cross-linked with glutaraldehyde. This sensor responded selectively to Hg^2+^ solutions of different concentrations (1 × 10^−5^ M to 0.1 M). The observed turbidity measured as absorbance varied between 1.05 and 1.75 units at a wavelength of 700 nm. The response is based on the interaction between the metal cations with the negative charges of the deprotonated dithiocarbamate functional group, which led to neutratization of the charges and thus to polymer shrinking. As a result, an increase in the turbidity of the sensing element due to a change in the refractive index between the hydrogel and the polymer microspheres occured. The changes in the turbidity of the sensing element were measured as absorbance using a conventional spectrophotometer.

## 1. Introduction

Recently, interest has been directed towards developing optical chemical sensors based on polymer swelling and shrinking [1–7]. Holtz *et al.* prepared a sensing element from a crystalline colloidal array of polymer spheres and a hydrogel that swells and shrinks in the presence of certain analytes [8]. Meanwhile, Liu *et al.* used a hydrogel containing various molecular recognition receptors that are subject to polymer swelling for the detection of different chemical species [9]. Some of these sensors involved use a variety of functional groups attached chemically to the backbone of polyvinylbenzyl chloride polymer. Seitz *et al.* developed an optical sensing element from lightly cross-linked chemically derivatized polymer microspheres with dimensions of a few micrometers. These microspheres were suspended in a hydrogel membrane which changed its volume reversibly in response to changes in analyte concentrations. This chemical sensing is based on the changes in the optical properties of the membrane that accompany swelling and shrinking. On swelling the refractive index of the microspheres becomes closer to that of the hydrogel, resulting in a decrease in the membrane turbidity [4,6,10].

In previous work, we developed a sensor in which polyvinylbenzyl chloride microspheres were aminated with ethylenediamine, and then converted to the respective dithiocarbamate by reacting the product with carbon disulfide. The resulting dithiocarbamate functionality was selected due to its formation of stable chelates with heavy metal ions of environmental concern, and to its negligible affinity towards alkali and alkaline earth metal ions typically present in real samples [2,11]. The response to metal cations is due to the neutralization of the negative charges on the deprotonated dithiocarbamate that lead to shrinking of the microspheres. This results in a change in the refractive index between the microspheres and the hydrogel membrane which can be measured as turbidity. This sensor has some limitations, particularly, due to its low sensitivity and long response time. To overcome these limitations, the sensing element was modified by using a partially hydrophilic copolymer and a different aminating group. This modification led to more substitution during the functionalization of the microspheres with dithiocarbamate.

In this work, partially hydrophilic polyvinylpyridine-polyvinylbenzyl chloride copolymer microspheres were aminated with ethanolamine and then transformed into the corresponding dithiocarbamate copolymer. The resulting microspheres were entrapped in a polyvinylalcohol membrane cross-linked with glutaraldehyde to form an optical sensing element. The latter demonstrated significant improvement in both dynamic range sensitivity and response time.

## 2. Experimental

### 2.1. Reagents

Analytical grade *N,N*-dimethylfomamide (DMF), ethanolamine, 2-propanol, carbon disulfide (CS_2_), polyvinyl alcohol, glutaraldehyde 8% by weight, mercuric chloride, lead nitrate, cadmium chloride, zinc chloride, sodium chloride, potassium chloride, calcium chloride, magnesium chloride, were purchased from Sigma-Aldrich Co. (USA). All solutions were prepared in deionized distilled water. Poly vinyl pyridine-poly vinyl benzyl chloride copolymer(5%) lightly crosslinked with divinylbenzene (2% mole) microspheres of 1–3 micrometer diameter was supplied by the Chemistry Department at the University of New Hampshire (USA).

### 2.2. Instruments

Absorption measurements related to turbidity were performed on a Perkin-Elmer Lambda 5 UV-visible spectrophotometer. The pH measurements were recorded on a Jenway pH meter (3,310) with a combination glass electrode and a tolerance of ±0.01 pH units. A Fourier transform infrared spectrophotometer (Nicolet Avatar 370DTGS) was used to obtain IR spectra.

### 2.3. Synthesis of the dithiocarbamate polymer

One gram of (polyvinylpyridine-polyvinylbenzyl chloride) copolymer 1 was soaked in few milliliters of dimethylformamide for several days and then immersed in 7.5 mL of ethanolamine (2) at room temperature and stirred for one week. The product 3 was washed several times with distilled water then the excess ethanol amine was removed under reduced pressure.

**Scheme 1.**
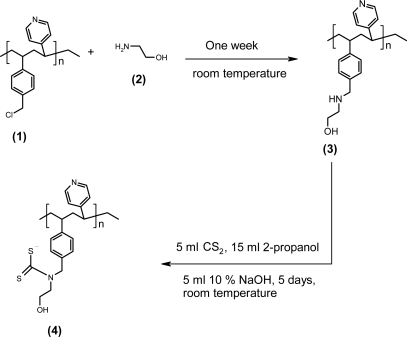
Synthesis of dthiocarbamate copolymer 4.

Next one gram of the aminated copolymer microspheres 3 was stirred with a mixture of 15 mL of 2-propanol, 5 mL CS_2_, and 20 mL DMF for one hour at room temperature. This was followed by the addition of 5 mL of 10% aqueous NaOH and the resulting solution was stirred for five days. The dithiocarbamate copolymer **4** was then filtered and washed several times with distilled water and dried under reduced pressure (Scheme 1).

### 2.4. Polymer capacity

The capacity of dithiocarbamate copolymer microspheres 4 towards metal ions was determined by initially soaking a 0.1 gram of the derivatized copolymer in 20 mL of aqueous 0.1 M HgCl_2_ and stirring overnight. Then, the copolymer was filtered and washed extensively with distilled water. The unadsorbed metal ions on the copolymer were determined by inductively coupled plasma (ICP). Then the adsorbed metal ions on the copolymer were calculated.

### 2.5. Optical measurements

The sensing element prepared as described previously was stuck on the inner sidewall of a cuvette which was then secured in the cell holder of a conventional Perkin Elmer spectrophotometer [2,6], such that the membrane was positioned in the path of the light beam. The solution in the cuvette was changed using a disposable pipette, starting with the lower concentrations of analyte and proceeding to the higher ones, with an interval of 10 minutes between each spectrum run. The response of the sensor to pH was performed starting with the lower pH and proceeding to the higher ones (2.0–12.6). The reproducibility of the sensor response was evaluated by cycling between blank and 0.1 M Hg^2+^ ions several times. Readings as turbidity absorbance at wavelength (700 nm) were taken after 10 minutes of introducing the solution in the cuvette. Between each reading, Hg^2+^ ions were eluted by a saturated solution of EDTA and then washed extensively with distilled water until a blank reading was obtained. The response time of the sensing element towards 0.1 M of Hg^2+^ ions was obtained by measuring the change in turbidity as absorbance with time, until a steady state was reached. After the sensor responded to metal cation, a saturated solution of EDTA was added, followed by basic buffer solution and finally washing successively with distilled water to regenerate the sensing element.

## 3. Results and Discussion

### 3.1. Characterization of the dithiocarbamate polymer

**Figure 1.**
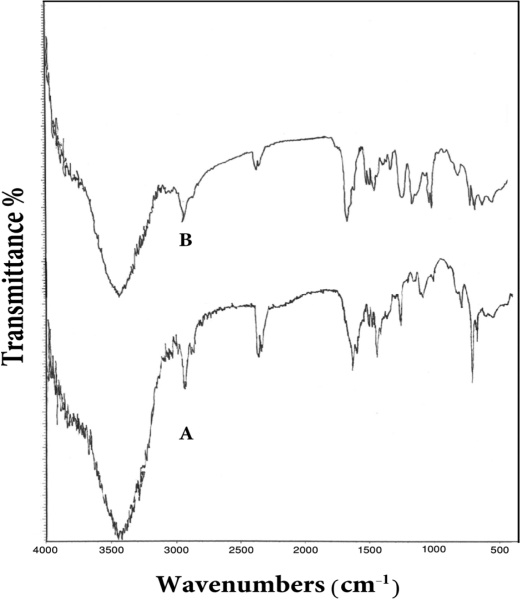
FTIR spectra: (A) Polyvinylpyridine-polyvinylbenzyl chloride copolymer. (B) Dithiocarbamate copolymer.

The introduction of the dithiocarbamate groups into the copolymer backbone was characterized by the disappearance of the C–Cl bond stretching at 800 cm^−1^ and 600 cm^−1^ and the appearance of peaks at ∼1,500 cm^−1^ which are related to the C-N vibration of CS_2_-NR_2_ bond and peaks between 1,200 cm^−1^ and 1,050 cm^−1^ which are related to the C=S vibration of the CSS bond (Figure 1).

Capacity studies showed that the amount of Hg^2+^ ions absorbed was 13.5 mmol per gram of the copolymer as opposed to 1.1 mmol per gram of the dithiocarbamate functionalized polyvinylbenzyl chloride polymer. The use of the copolymer improved significantly the sensitivity and increased the dynamic range of the sensor due to the presence of the pyridine moiety in the polymer backbone. The latter acted to space out the polymer reducing steric hindrance and thus resulting in more substitution.

### 3.2. Sensor evaluation

**Figure 2.**
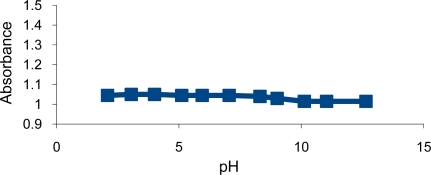
Turbidity absorbance of sensing element for 0.001 M Hg^2+^ at different pHs.

When the sensing element was examined with different pH buffer solutions (2.0–12.6), insignificant changes in absorbance were observed. This probably results from the presence of both basic (amine groups) and acidic groups (CS_2_H) situated on the dithiocarbamate copolymer 4. The nitrogen (hard base) and the sulfur (soft base) were both protonated at low pH, where positive charges on nitrogen repel each other causing the polymer to be in a swollen state. As the pH is raised, the sulfur on the dithiocarbamate group becomes deprotonated and so the resulting negative charges repel each other causing the polymer to stay in a swollen state. This behavior does not lead to any significant and observable shrinking process, and thus no detectable changes in absorbance (Figure 2).

**Figure 3.**
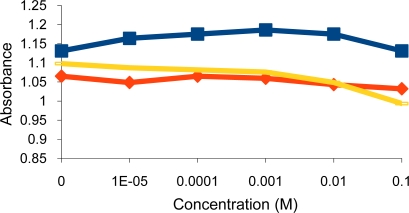
Turbidity absorbance of sensing element *vs.* concentration of Zn^2+^ ( 
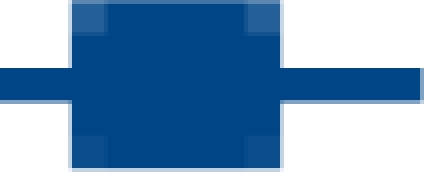
), Ni^2+^ ( 
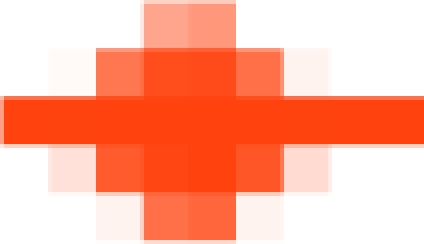
) and Cr^3+^ ( 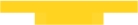) ions.

**Figure 4.**
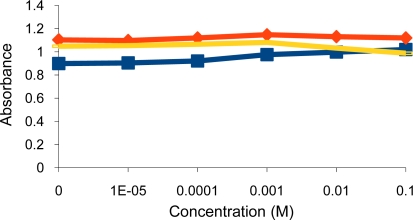
Turbidity absorbance of sensing element *vs.* concentration of Cu^2+^ ( 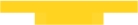), Cd^2+^ ( 
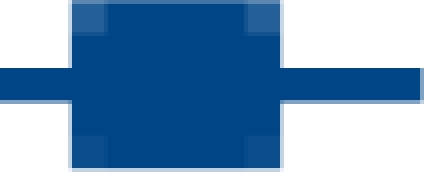
) and Pb^2+^ ( 
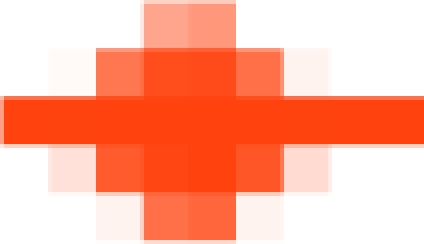
) ions.

**Figure 5.**
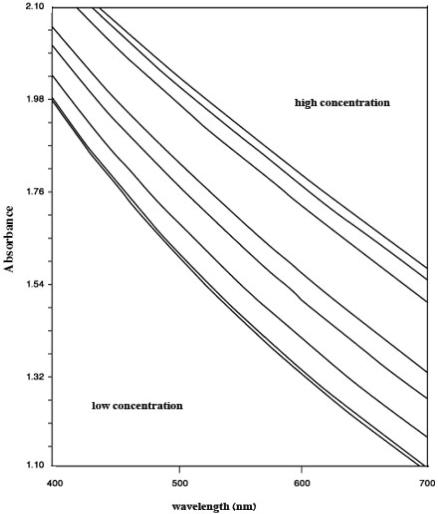
Variation of absorbance of sensing element *vs.* wavelength of different Hg^2+^ ion concentrations.

**Figure 6.**
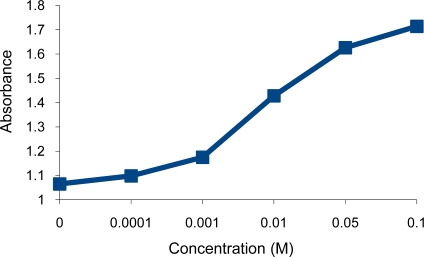
Turbidity absorbance of sensing element *vs.* concentration of Hg^2+^ ions.

Surprisingly, the deprotonated dithiocarbamate copolymer showed no optical response towards tested heavy metal cations (Ni^2+^, Cu^2+^, Cr^3+^, Pb^2+^, Zn^2+^, Cd^2+^), while it showed a very high affinity towards the soft metal cation Hg^2+^ (Figures 3,4). The response of our sensing element towards Hg^2+^ was tested by using different aqueous solutions with concentrations ranging from 1 × 10^−5^ M to 0.1 M Hg^2+^. At this concentration range, the turbidity increased from 1.05 to 1.75 measured as absorbance (Figures 5,6), as opposed to 0.74 to 0.86 in the previously tested dithiocarbamate polymer derived from polyvinylbenzyl chloride [2].

The increase in turbidity with Hg^2+^ concentration is probably due to the formation of a complex between Hg^2+^ and the deprotonated dithiocarbamate groups, thus causing the polymer microspheres to shrink due to neutralization of the negative charges on the sulfur atoms. This shrinkage increased the difference in the refractive index between the hydrogel and the microspheres, which resulted in an increase in the turbidity of the sensing; this was measured as absorbance.

Thus, the use of polyvinylpyridine-polyvinylbenzyl chloride copolymer instead of polyvinylbenzyl chloride polymer improved the sensitivity and increased the dynamic range. The presence of the pyridine group in the polymer backbone apparently increased the space between groups in the polymer backbone. This decreased the steric hindrance, and resulted in more substitution.

**Figure 7.**
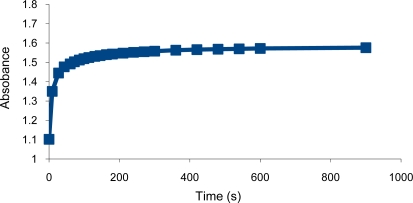
Response time of the sensing element towards Hg^2+^ ions.

The response time of the sensing element toward Hg^2+^ ions was obtained by recording the change in turbidity as absorbance of 0.1 M Hg^2+^ at 700 nm *vs.* time. The absorbance increased with time until it reached a steady state. The response time was significantly shorter with the copolymer than that obtained with poly vinyl benzyl chloride. Thus, the copolymer took 30 seconds to reach 90% response while the polymer needed 10 minutes under the same conditions (Figure 7) [2]. This fast response is probably due to the presence of the hydrophilic pyridine group in the copolymer which enhanced the movement of the analyte and solvent across the microspheres during the shrinking process.

**Figure 8.**
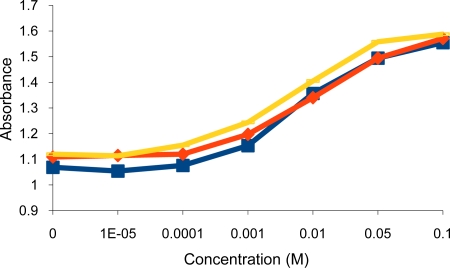
Turbidity absorbance of sensing element *vs.* concentration of Hg^2+^ after one ( 
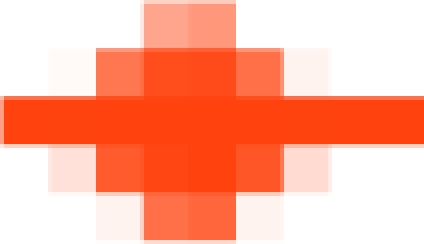
), two ( 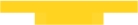) and three ( 
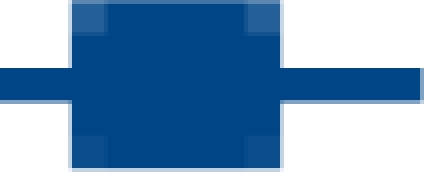
) weeks.

The reproducibility of the sensing element was examined by taking the change in turbidity as absorbance of a 0.1 M Hg^2+^ aqueous solution at 700 nm. The absorbance for the blank (1.109) and 0.1 M Hg^2+^ (1.648) stayed almost constant during the ten runs, and according to the standard deviation calculations (4.714 × 10^−4^), this sensing element is highly reproducible. The stability of the sensor was examined by measuring the response to Hg^2+^ during several weeks; one run was taken every week. This sensing element was found to be stable for several weeks during which it gave a positive response towards Hg^2+^ ions (Figure 8).

Both the reproducibility and stability of the sensor are related to the utilization of the very small particles (1–3 μm) of the copolymer which provided mechanical stability and flexibility during multiple swelling and shrinking cycles. This result is an indication of the high stability of the dithiocarbamate functional group under the conditions of the experiment.

**Figure 9.**
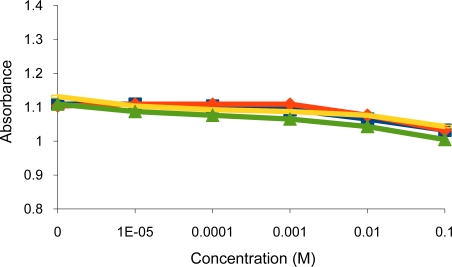
Turbidity absorbance of sensing element *vs.* concentration of alkali; Na^+^ ( 
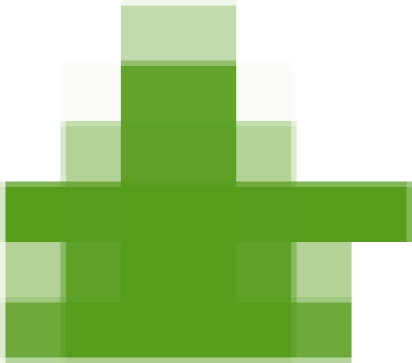
), K^+^( 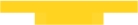) and alkaline earth; Mg^2+^( 
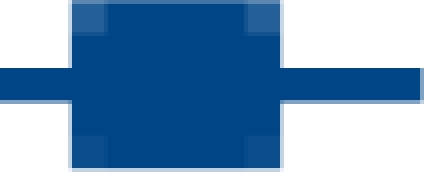
), Ca^2+^( 
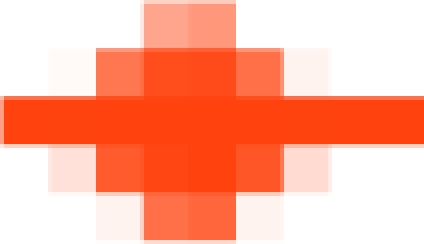
) metal ions.

As expected the dithiocarbamate copolymer showed no response towards alkali and alkaline earth metal cations (Figure 9).

**Figure 10.**
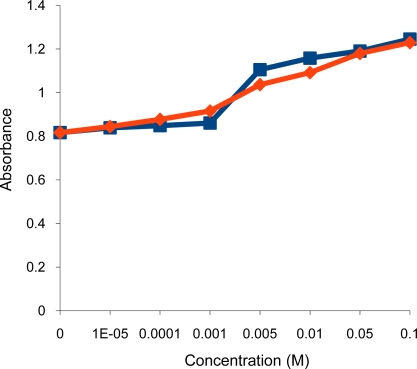
Turbidity absorbance of sensing element *vs.* concentration of Hg^2+^ in tap ( 
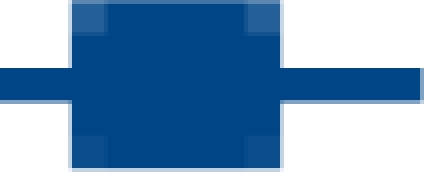
) and distilled water ( 
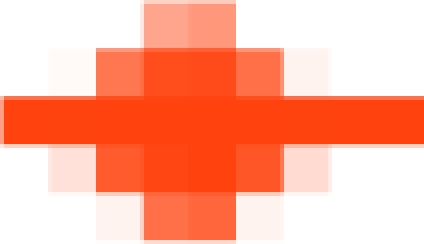
).

This is because the sulfur atoms of the dithiocarbamate group are soft ligands and do not interact with hard metal cations (alkali and alkaline earth metals). The presence of these metal cations will not affect the optical properties of the sensor, this is demonstrated by the significant similarity in response towards Hg^2+^ ions in both tap and distilled water (Figure 10). Thus, this sensor has the potential to be applied to real samples.

## 4. Conclusions

A new improved optical chemical sensor based on swellable polymer microspheres has been developed using a dithiocarbamate functional group covalently bonded to a backbone of polyvinylpyridine-polyvinylbenzyl chloride copolymer. This gave better performance compared to previously prepared sensors. It showed better sensitivity, reproducibility, stability and shorter response time towards Hg^2+^ions. In addition, no significant response to the heavy metal ions (Ni^2+^, Cu^2+^, Cr^3+^, Pb^2+^, Zn^2+^, Cd^2+^) and the alkali and alkaline earth metal ions was detected.
